## [Peer Review File · Nature Communications]

Reviewers' comments:

Reviewer #1 (Remarks to the Author):

The authors synthesized and characterized an iron metal-ligand nanocluster. The spectroscopy analysis including ^{57}Fe Mössbauer spectra, in situ FT-IR, XAS, XMCD indicates the molecule may have an extended delocalized electronic structure. It is very interesting that it seemed that there are two electrons fully delocalized among the six Fe centers. This large mixed-valence delocalized system is rarely seen in coordination compounds. Some discussions about the electronic structure are not very clear and need be addressed before publication.

1. Based on the Mössbauer spectroscopic measurements, the authors concluded that the electron hopping rate is fast at room temperature and may be frozen and distributed over the disparate B-site Fe ions at low temperature. This changed electronic structure will produce considerable spectroscopic signals. Although FT-IR is too fast to observe the bonding change of fully delocalized and localized electronic structure, is it possible to find other appropriate clear characterization to see this change?
2. The fully delocalized mixed-valence state at room temperature may be changed by the physical or other stimuli, for example, high magnetic field. Could it be found in this molecule?
3. EA is a fundamental characterization and should be provided in the manuscript.
4. The spin density of site-B Fe ions by DFT calculations was provided in SI but only average values. Model calculation on spin distribution will be helpful to understand the localization of hopping electrons. It is better to give spin density of the individual site-B Fe ions.

Reviewer #2 (Remarks to the Author):

This paper describes an Fe_{14} clusters with delocalized valence electrons. Structure and physical analyses have been reasonably done. I think this paper can be accepted after considering the following points.

- 1) Mössbauer spectra suggest localized and delocalized valence electrons upon temperature variation. However, this can be claimed only in the Mössbauer time window. I think the valence

electrons are never localized in all temperature range measured. The title of “delocalized -localized transition” is not appropriate.

2) The valence electron on a high-spin (HS) Fe(II) is suggested to be transferred (or delocalized) to a HS Fe(III) ions through d- π orbital of a low-spin (LS) Fe(III) ion and $p\pi^*$ orbitals of two cyanide ions. This seems not to be probable because π and π -back bonding interactions of HS Fe(II and III) ions with the cyanide groups seems to be very weak. Is there any theoretical (DFT) calculation which supports their hypothesis?

3) I wonder the Fe₁₄ shows an intervalence band in its uv-vis (or near infrared) spectrum. Analyses of the IVCT band give information about the valence electron delocalization (for example, H_{ab} and α^2 values).

4) The magnetization curve in Figure 4b should be simulated by the Brillouin function with anisotropic terms.

Reviewer #3 (Remarks to the Author):

The manuscript of Huang et al. describes the properties of a mixed valence complex comprised of 14 iron ions forming a cube. 8 iron ions are located at the corners of the cube and are valence-localized Fe(II) l.s. The remaining 6 iron ions are located at the center of the cubic faces and exhibit formally 4 Fe(III) h.s. and 2 Fe(II) h.s. ions. Using techniques working on different time scales, it is shown that these 6 h.s. ions form a class II mixed valence system according to the Robin&Day classification. I.e., there is a temperature-dependent electron hopping between the 6 iron ions and not a quantum-mechanical delocalization (class III). The electron hopping through the cyanide bridges is correlated with a ferromagnetic coupling between the six iron ions. From this scientific description, the requirements for publication in Nature Communications are fulfilled. However, before publication in Nature Communications can be recommended there must be major revision of the manuscript.

1. The use of nomenclature used in some instances is not appropriate which can be due to the interdisciplinary group of chemists and physicists or simply due to overselling. The molecule described herein is a transition metal complex, i.e. metal center coordinated by ligands. The ligands can be simple inorganic anions as Cl⁻ or organic molecules. There is no need to rephrase known kind of compounds in coordination chemistry by trying to establish phrases as metal-organic materials (MOMs). Moreover, a complex (with or without organic ligands) is a typical inorganic compound. It is misleading and inappropriate to just phrase extended solid-state compounds as oxides as inorganic. Moreover, phrases as nanoclusters are overselling and not needed. Nano-sized complex is a neutral but well describing phrase.

2. lines 55-59: The concomitant occurrence of electron transfer and electron exchange is well established in physics (Zener Phys. Rev. 1951, Anderson&Hasegawa Phys. Rev. 1955) and chemistry (Blondin&Girerd Chem. Rev. 1990) literature. However, despite these fundamental references, references to publications describing the properties of mixed valence complexes of either hopping or delocalized electronic structure are missing (e.g. Wieghardt&Chaudhuri Chem. Comm. 1989, Wieghardt&Solomon JACS 1996, Wieghardt JACS 1999, Long Nature Chem. 2010).

3. IUPAC: A ligand abbreviation in a formula of a complex has to be written in brackets: (Tp) instead Tp.

4. The temperature dependence of the Moessbauer spectra demonstrate that this complex is of class II mixed valence of the Robin&Day nomenclature, i.e. a temperature-dependent electron transfer (hopping). Neither this classification nor even this reference (Adv. Inorg. Chem. Radiochem. 1967) is provided. I am a little bit confused by the Moessbauer simulations (Fig. 2a,b and Table S4). There is an example in Wieghardt JACS 1999 on the temperature dependence of the Moessbauer spectra in a class II mixed valence Fe(II)Fe(III) complex. In that complex, the Moessbauer spectra can be simulated with localized Fe(II) and Fe(III) at low temperatures, while a quadrupole doublet of mean isomer shift and quadrupole splitting grows in with increasing temperature, which corresponds to the temperature dependent percentage of complex with fast electron transfer (ET). No line-broadening effects (coalescence) were observed in the intermediate temperature range, where both species - slow and fast ET - occur. Is a simulation with such a model possible?

Instead, the authors used an electron hopping relaxation model, which is a good choice. The authors provide values for isomer shift and quadrupole splitting for static Fe(II) and Fe(III) and not of fast ET species of intermediate values. The authors should describe this model in more detail in the Supp. Inf. and should also provide all simulated Moessbauer spectra and not only some selected temperatures.

5. lines 137-139: A unique asymmetric doublet is not indicative for electron hopping as in the example above, a unique symmetric doublet was observed for fast-ET species. Thus, it should be phrased 'a unique doublet ...'.

6. lines 149-151 and related in many parts of the manuscript: This complex exhibits a temperature-dependent electron hopping that can be described by an Arrhenius law. Thus, there is no transition from a frozen to a hopping phase/regime (this was also confirmed by the Cp measurements). Instead, the hopping becomes faster with increasing temperature. Thus, the time scale of the used experimental technique determines the apparent behavior. This time scale can be faster (appears as frozen), it can be slower (appears as averaged), or it can be in the time window of the electron hopping so that a temperature-dependent signal can be observed (from an apparent static

to fast behavior). However, the apparent static behavior is not a frozen situation, it is just slower than the time scale of the technique.

7. lines 171-172: No, it is not delocalized (class III), it is faster than the MB time window. It is still a hopping between existent states.

8. lines 182-183: As in point 7, there is no quantum tunneling.

9. lines 292: Again, this is not a delocalized mixed valence complex (class III), it is mixed complex with a temperature-dependent electron hopping (class II).

Response to reviewer 1:

Comments:

The authors synthesized and characterized an iron metal-ligand nanocluster. The spectroscopy analysis including ^{57}Fe Mössbauer spectra, in situ FT-IR, XAS, XMCD indicates the molecule may have an extended delocalized electronic structure. It is very interesting that it seemed that there are two electrons fully delocalized among the six Fe centers. This large mixed-valence delocalized system is rarely seen in coordination compounds. Some discussions about the electronic structure are not very clear and need to be addressed before publication.

Response: We thank the reviewer for their positive comments on our work and valuable suggestions concerning the manuscript. The manuscript was revised following all the points raised by the reviewer and the point-by-point responses are given below.

1. Based on the Mössbauer spectroscopic measurements, the authors concluded that the electron hopping rate is fast at room temperature and may be frozen and distributed over the disparate B-site Fe ions at low temperature. This changed electronic structure will produce considerable spectroscopic signals. Although FT-IR is too fast to observe the bonding change of fully delocalized and localized electronic structure, is it possible to find other appropriate clear characterization to see this change?

Response: In light of reviewer's comments, we carefully considered the timescales of the characterization techniques. The spectroscopic studies (including FT-IR, XAS) revealed a localized electronic structure for $[\text{Fe}_{14}]$. In the revised version, we also include the UV-Vis-NIR spectra (Supplementary Figure 3) and the additional analysis was consistent with other spectroscopic characterizations. The time domain for the observed phenomenon is suitable for Mössbauer spectra reported here.

Furthermore, we considered other suitable techniques and found that the muon spin relaxation could be a suitable alternative analysis because the timescale of the muon spin relaxation is approximately 10^{-7} s. We believe it will provide important complementary information. We are now applying for the measurement time in ISIS but an extended wait is anticipated to obtain beam time at the center. Therefore, we are unable to include this data in our revised manuscript. Notably, as the evidence presented

in the manuscript clearly supports our conclusion, we believe the additional information from muon spin relaxation will not alter current conclusion.

2. The fully delocalized mixed-valence state at room temperature may be changed by the physical or other stimuli, for example, high magnetic field. Could it be found in this molecule?

Response: We have carefully considered that electron hopping may be altered under external stimuli. We investigated the effect of light irradiation on mixed-valence state of [Fe₁₄] as the control of the electronic structure by light is an area of ongoing research interest in our group. It has been reported by multiple groups, including us, that several cyanide-bridge coordination polymers undergo photoinduced charge transfer (Y.-S. Meng et al., *Angew. Chem. Int. Ed.*, **2018**, 57, 12216), enabling the modulation of the electronic and magnetic properties of the compounds. Therefore, we performed photo-magnetometry on [Fe₁₄] with laser irradiation centered at 405 nm (corresponding to the LMCT band of [Fe₁₄]) to determine if the electronic structure and/or electron hopping can be controlled via the trapping of light-induced electron-transferred metastable states. Unfortunately, this photo effect was not observed in our preliminary experiment. However, switching behavior may be observed under different experimental conditions or by chemical modification of the [Fe₁₄] parent complex.

Unfortunately, no high magnetic field equipment is installed on our Mössbauer spectrometer. Therefore, we are pursuing investigations on magnetic field effects in future in collaboration with other research groups, who can measure Mössbauer spectra under magnetic field.

We will attempt to expand the related study as suggested by the reviewer. Thank you for your valuable comments, which really inspire our imagination about future investigations on our [Fe₁₄] complex.

3. EA is a fundamental characterization and should be provided in the manuscript.

Response: We assume that “EA” mentioned by the reviewer is the abbreviation for “elemental analysis,” and this analysis (CHN) is provided for [Fe₁₄] in the synthesis section. In addition, the Fe content was analyzed through ICP-AES. For reference, the analysis for [Fe₁₄] is provided here. Anal. Calcd. for C₁₀₈H₁₂₈B₈Fe₁₄N₇₂O₁₂S₆: C, 35.18;

H, 3.50; N, 27.35. Found: C, 35.12; H, 3.52; N, 27.40. The amount for Fe (21.27 wt%) is also in good agreement with the calculated value of 21.20 wt%.

4. The spin density of site-B Fe ions by DFT calculations was provided in SI but only average values. Model calculation on spin distribution will be helpful to understand the localization of hopping electrons. It is better to give spin density of the individual site-B Fe ions.

Response: In light of the reviewer's comments, we provided the spin density (Supplementary Figure 6, SI) of each Fe ion from both A and B sites. The DFT calculations show that the spins are approximately evenly distributed over the 6 B-site Fe centers with a small portion on the 8 A-site Fe centers. The calculated electronic structure suggests either a delocalized ground state or a mixture of multiple quasi-degenerate electronic configurations. Experimental results show that the [Fe₁₄] cluster is assigned to a Class II mixed valence compound, which is consistent with the latter situation.

Response to reviewer 2:

Comments:

This paper describes an Fe₁₄ clusters with delocalized valence electrons. Structure and physical analyses have been reasonably done. I think this paper can be accepted after considering the following points.

Response: We thank the reviewer for the evaluation of this work and very valuable suggestions aiming at the enhanced quality of manuscript. The manuscript was revised following all the points raised by the reviewer and the point-by-point responses are given below.

1) Moessbauer spectra suggest localized and delocalized valence electrons upon temperature variation. However, this can be claimed only in the Moessbauer time window. I think the valence electrons are never localized in all temperature range measured. The title of “delocalized -localized transition” is not appropriate.

Response: We thank the reviewer for the constructive suggestion. Considering the different timescales of common spectroscopic techniques, we revised all relevant instances concerning the phrase “localized” throughout the manuscript. The title was also changed to “Temperature dependence of spherical electron transfer in a nanosized [Fe₁₄] complex.”

2) The valence electron on a high-spin (HS) Fe(II) is suggested to be transferred (or delocalized) to a HS Fe(III) ions through d- π orbital of a low-spin (LS) Fe(III) ion and $\pi\pi^*$ orbitals of two cyanide ions. This seems not to be probable because π and π -back bonding interactions of HS Fe(II and III) ions with the cyanide groups seems to be very weak. Is there any theoretical (DFT) calculation which supports their hypothesis?

Response: Thank you for the insightful comment on the electron pathway. In light of the reviewer’s comments, we examined the spin density (Supplementary Figure 6, SI) of each Fe atom from both A and B sites. A small portion of spin densities could be found on the A sites, showing that electrons are partially delocalized through the d π orbital of a low-spin (LS) Fe(II) ion and $\pi\pi^*$ orbitals of two cyanide ions, which supports the hypothesis. However, the extent of such delocalization is weak. It should

be noted that strong delocalization of the unpaired electrons is expected to induce a large magnetic interaction. The observed weak magnetic coupling indicates that the delocalization pathway should not be an efficient one.

3) I wonder the Fe₁₄ shows an intervalence band in its uv-vis (or near infrared) spectrum. Analyses of the IVCT band give information about the valence electron delocalization (for example, H_{ab} and α^2 values).

Response: In light of the referee's suggestion, we provided the UV-Vis-NIR spectra in Suppl. Fig. 3. The wide peak around 11500 cm⁻¹ is assigned to the optical intervalence charge-transfer (IVCT) band. According to the Robin & Day classification, the full width half maximum, or half-bandwidth, ($\Delta\bar{\nu}_{1/2}$) at the optical IVCT transition is used as a simplified criterion for distinguishing between Class II and III valence delocalization. However, the IVCT band in [Fe₁₄] is rather complicated compared to typical mixed valence complexes because it consists of two components, *i.e.*, adjacent IVCT (Fe^{II}-CN-Fe^{III}) and remote IVCT (Fe^{II}-NC-Fe^{II}-CN-Fe^{III}) bands (for examples, see: Oshio et al, *Inorg. Chem.* **47**, 6106–6108 (2008); *Chem. Eur. J.*, **6**, 2523–2530 (2000); *Chem. Eur. J.* **9**, 3946–3950 (2003)). Therefore, to obtain information about the valence electron delocalization (H_{ab} and α^2 values), we have to deconvolute the broad IVCT band into two individual peaks. Here, a conclusive analysis is hindered due to the fact that the contribution of each IVCT band to the broad IVCT absorption is not clear. Therefore, we avoided going detailed discussion on valence electron delocalization but have provided the UV-Vis-NIR spectra in the revised supplementary information.

4) The magnetization curve in Figure 4b should be simulated by the Brillouin function with anisotropic terms.

Response: In light of the reviewer's suggestion, the magnetization curve in Figure 4b has been simulated with the Brillouin function (green line). To explore this, we explored two models: (1) a $S = 14$ spin ground state with weak axial zero field splitting and (2) Heisenberg exchange. We found that inclusion of weak axial zero field splitting does not fit the magnetization curve particularly well and is not consistent with high frequency EPR simulations. However, the inclusion of a weak ferromagnetic exchange

interaction acting between bridged high spin sites is found to reproduce the measured magnetization curve. The exchange model neglects the double-exchange (DE) component to the Hamiltonian present in Class II systems, however such a detailed DE model results in over-parameterization. Hence, the use of the Heisenberg model sufficiently indicates how the measured curve is consistent with weak ferromagnetic exchange coupling within $[\text{Fe}_{14}]$. The simulation details were provided in Methods.

Response to reviewer 3:

Comments:

The manuscript of Huang et al. describes the properties of a mixed valence complex comprised of 14 iron ions forming a cube. 8 iron ions are located at the corners of the cube and are valence-localized Fe(II) l.s. The remaining 6 iron ions are located at the center of the cubic faces and exhibit formally 4 Fe(III) h.s. and 2 Fe(II) h.s. ions. Using techniques working on different time scales, it is shown that these 6 h.s. ions form a class II mixed valence system according to the Robin&Day classification. i.e., there is a temperature-dependent electron hopping between the 6 iron ions and not a quantum-mechanical delocalization (class III). The electron hopping through the cyanide bridges is correlated with a ferromagnetic coupling between the six iron ions. From this scientific description, the requirements for publication in Nature Communications are fulfilled. However, before publication in Nature Communications can be recommended there must be major revision of the manuscript.

Response: We greatly appreciate the reviewer's positive comments on this work. Considering the constructive suggestions, we carefully revised the manuscript accordingly and the point-by-point responses are given below.

1. The use of nomenclature used in some instances is not appropriate which can be due to the interdisciplinary group of chemists and physicists or simply due to overselling. The molecule described herein is a transition metal complex, i.e., metal center coordinated by ligands. The ligands can be simple inorganic anions as Cl⁻ or organic molecules. There is no need to rephrase known kind of compounds in coordination chemistry by trying to establish phrases as metal-organic materials (MOMs). Moreover, a complex (with or without organic ligands) is a typical inorganic compound. It is misleading and inappropriate to just phrase extended solid-state compounds as oxides as inorganic. Moreover, phrases as nanoclusters are overselling and not needed. Nano-sized complex is a neutral but well describing phrase.

Response: In light of the reviewer's suggestion, we have altered a number of phrases to avoid overselling. The changed phrases were marked with yellow background.

2. lines 55-59: The concomitant occurrence of electron transfer and electron exchange is well established in physics (Zener Phys. Rev. 1951, Anderson&Hasegawa Phys. Rev. 1955) and chemistry literature. However, despite these fundamental references, references to publications describing the properties of mixed valence complexes of either hopping or delocalized electronic structure are missing (e.g., Wieghardt&Chaudhuri Chem. Comm. 1989, Wieghardt&Solomon JACS 1996, Wieghardt JACS 1999, Long Nature Chem. 2010).

Response: In light of the referee's comment, we have included citations to additional important references in the field of mixed-valence.

3. IUPAC: A ligand abbreviation in a formula of a complex has to be written in brackets: (Tp) instead Tp.

Response: We added the brackets of ligand abbreviation in the formula.

4. The temperature dependence of the Moessbauer spectra demonstrate that this complex is of class II mixed valence of the Robin&Day nomenclature, i.e., a temperature-depending electron transfer (hopping). Neither this classification nor even this reference (Adv. Inorg. Chem. Radiochem. 1967) is provided.

Response: Thanks to the reviewer's suggestion, we have added additional description of the Class II mixed valence of the Robin & Day classification. The important reference (Adv. Inorg. Chem. Radiochem. 1967) is also cited in ref.26.

I am a little bit confused by the Moessbauer simulations (Fig. 2a,b and Table S4). There is an example in Wieghardt JACS 1999 on the temperature dependence of the Moessbauer spectra in a class II mixed valence Fe(II)Fe(III) complex. In that complex, the Moessbauer spectra can be simulated with localized Fe(II) and Fe(III) at low temperatures, while a quadrupole doublet of mean isomer shift and quadrupole splitting grows in with increasing temperature, which corresponds to the temperature dependent percentage of complex with fast electron transfer (ET). No line-broadening effects (coalescence) were observed in the intermediate temperature range, where both species - slow and fast ET - occur. Is a simulation with such a model possible?

Instead, the authors used an electron hopping relaxation model, which is a good choice. The authors provide values for isomer shift and quadrupole splitting for static Fe(II) and Fe(III) and not of fast ET species of intermediate values. The authors should describe this model in more detail in the Supp. Inf. and should also provide all simulated Mössbauer spectra and not only some selected temperatures.

Response: As the reviewer suggested, we have included a detailed explanation of the electron hopping relaxation model in the Supp. Inf. in addition to all the simulated Mössbauer spectra. The fast and slow ET model of Wieghardt did not correlate with our results, and it seems to be an unsuitable model for application to a static Class II compound. According to the reference reported by Wieghardt (JACS 1999, 121, 2193.), the Mössbauer spectra of the Class II mixed-valence [LFeCoFeL] complex were simulated by assuming localized Fe^{2+} and Fe^{3+} components and another Fe component (maybe $\text{Fe}^{2.5+}$ state) which is the delocalized species with mean IS/QS values. The spectrum at the highest temperature was described as only the delocalized (fast ET) doublet, while lower-temperature spectra were assigned to the two localized (slow ET) doublets. The intermediate spectra were attributed to a thermal distribution among the three Fe states. However, such an interpretation is unlikely to be appropriate because a Class II Fe^{2+} - Fe^{3+} compound has a double-well potential originated in Fe^{2+} and Fe^{3+} , which affords only the two states around the ground level and not three states ($\text{Fe}^{2.5+}$ is just an excited state). Assuming such a double-well potential, the excited $\text{Fe}^{2.5+}$ state (*i.e.*, delocalized state) is never populated at higher temperatures, which does not correspond to the Wieghardt's result. The result indicates a transition from Class II to class III on heating. Since the details of the results (*e.g.*, Mössbauer parameters in the whole temperature range) are not available from the reference, we are not able to discuss this further. On the other hand, the electron hopping relaxation model is based on a two component (Fe^{2+} and Fe^{3+}) assumption and does not include the excited $\text{Fe}^{2.5+}$ states. A fast exchange between two states often leads to a line broadening in spectroscopic signals, which is a very popular phenomenon observable in NMR and ESR and is well-known as "exchange broadening/narrowing" or "motional broadening/narrowing." Actually, the spectral profiles of $[\text{Fe}_8^{57}\text{Fe}_6]$ around 204–265 K broadened remarkably compared with those at the other temperatures. Curve fittings

using the electron hopping relaxation model satisfactorily reproduced the obtained spectra. Here, we have provided the full zero-field ^{57}Fe Mössbauer spectra at all investigated temperatures. (Supplementary Fig. 10)

Supplementary Figure 10. Zero-field ^{57}Fe Mössbauer spectra at all investigated temperatures.

5. lines 137-139: A unique asymmetric doublet is not indicative for electron hopping as in the example above, a unique symmetric doublet was observed for fast-ET species. Thus, it should be phrased ‘a unique doublet ...’.

Response: As the reviewer suggested, we deleted word “asymmetric” in the sentence.

6. lines 149-151 and related in many parts of the manuscript: This complex exhibits a temperature-dependent electron hopping that can be described by an Arrhenius law. Thus, there is no transition from a frozen to a hopping phase/regime (this was also confirmed by the C_p measurements). Instead, the hopping becomes faster with increasing temperature. Thus, the time scale of the used experimental technique determines the apparent behavior. This time scale can be faster (appears as frozen), it can be slower (appears as averaged), or it can be in the time window of the electron hopping so that a temperature-dependent signal can be observed (from an apparent static to fast behavior). However, the apparent static behavior is not a frozen situation, it is just slower than the time scale of the technique.

Response: We have altered these parts according to the reviewer’s comment.

7. lines 171-172: No, it is not delocalized (class III), it is faster than the MB time window. It is still a hopping between existent states.

Response: We changed this sentence in light of the reviewer’s comment.

8. lines 182-183: As in point 7, there is no quantum tunneling.

Response: We deleted the discussion of quantum tunneling.

9. lines 292: Again, this is not a delocalized mixed valence complex (class III), it is mixed complex with a temperature-dependent electron hopping (class II).

Response: We have changed this sentence in accord with the comment.

REVIEWERS' COMMENTS:

Reviewer #1 (Remarks to the Author):

The authors provided more experimental data and confirmed the compound is a class II mixed valence system. I would like to suggest the acceptance of the manuscript as the present form.

Reviewer #2 (Remarks to the Author):

The authors revised their manuscript to meet the criticisms raised by the reviewers. I think the paper can be accepted.

Reviewer #3 (Remarks to the Author):

The authors have seriously revised their manuscript according to the issues raised by the reviewers. Therefore, publication of the revised manuscript is recommended.

Reviewer #1 (Remarks to the Author):

The authors provided more experimental data and confirmed the compound is a class II mixed valence system. I would like to suggest the acceptance of the manuscript as the present form.

Reviewer #2 (Remarks to the Author):

The authors revised their manuscript to meet the criticisms raised by the reviewers. I think the paper can be accepted.

Reviewer #3 (Remarks to the Author):

The authors have seriously revised their manuscript according to the issues raised by the reviewers. Therefore, publication of the revised manuscript is recommended.

Response: We would like to thank all reviewers for the positive comment on this work.