# Supplementary material for: Temperature dependence of spherical electron transfer in a nanosized [Fe14] complex
Source: Nat Commun. 2019 Dec 3;10:5510. doi: 10.1038/s41467-019-13279-y (PMC6890645; doi:10.1038/s41467-019-13279-y)
Supplement: Supplementary file 1 — Supplementary Information [file 41467_2019_13279_MOESM1_ESM.pdf]

Supplementary Information for

Temperature dependence of spherical electron transfer in a nanosized [Fe_{14}] complex

Huang et al.

Correspondence to: wudy@cczu.edu.cn; sato@cm.kyushu-u.ac.jp.

Contents

Supplementary Fig. 1 | Thermogravimetric data of **[Fe_{14}]**.

Supplementary Fig. 2 | Crystal packing diagram at different temperatures.

Supplementary Fig. 3 | The electronic absorption spectra of **[Fe_{14}]** in solid state.

Supplementary Fig. 4 | *In-situ* infrared spectra of [**Fe_{42}**] recorded at different temperatures.

Supplementary Fig. 5 | Kβ emission spectroscopy and high-resolution K-edge XANES.

Supplementary Fig. 6 | Optimized atomic structure and spin density of **[Fe_{14}]**.

Supplementary Fig. 7 | X-ray magnetic circular dichroism measurements.

Supplementary Fig. 8 | Field-dependent magnetic data.

Supplementary Fig. 9 | High-frequency electron paramagnetic resonance spectra.

Supplementary Fig. 10 | Full zero-field ^{57}Fe Mössbauer spectra.

Supplementary Fig. 11 | Schematic demonstration of electron hopping relaxation model.

Supplementary Table 1 | The crystal data and results of the structure determination.

Supplementary Table 2 | The selected bond distances (Å) and angles (°) for **[Fe_{14}].**

Supplementary Table 3 | The Mössbauer fitting parameters for **[Fe_{14}].**

Supplementary Table 4 | The Mössbauer fitting parameters for **[$Fe_8{}^{57}Fe_6$]**.

Supplementary Method

Supplementary References

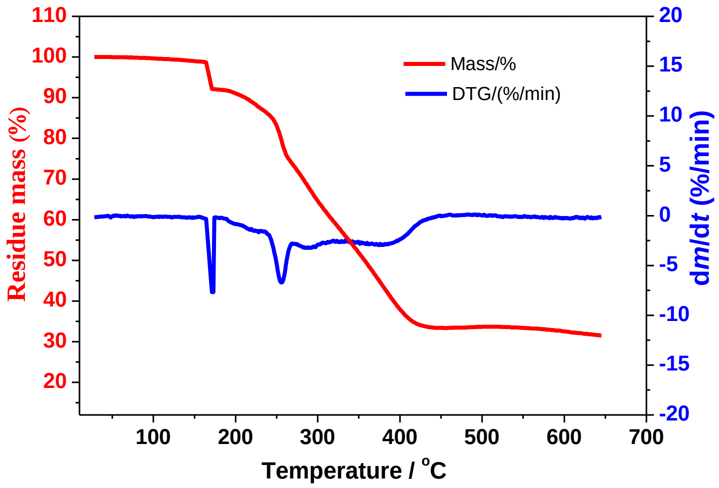

Supplementary Fig. 1 | Thermogravimetric data of [Fe_{14}].

a

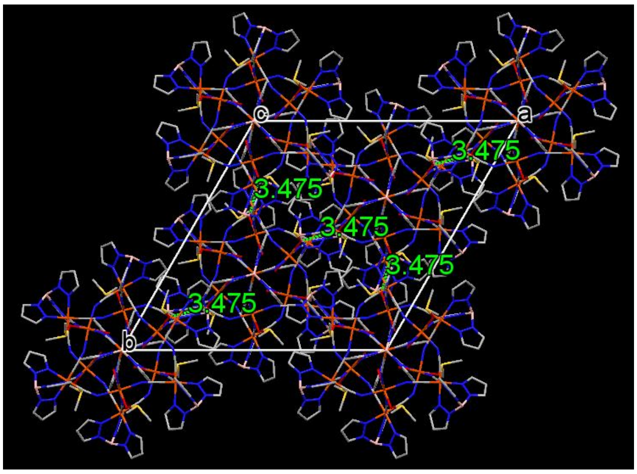

@ 25K

b

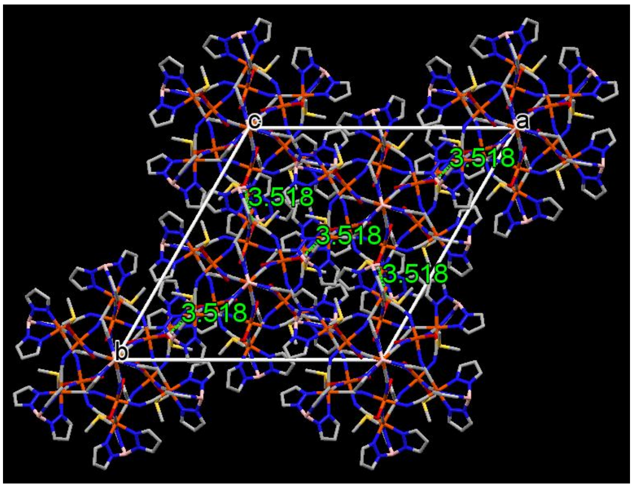

@ 123 K

c

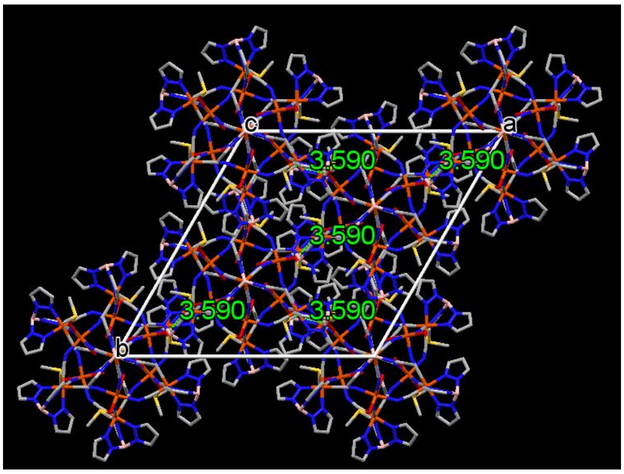

@ 298 K

Supplementary Fig. 2 | Crystal packing diagram. a, at 25 K, b, at 123 K, and c, at 298 K for **[Fe_{14}]** denoting the O···O intermolecular contact from the DMSO solvate ligand, respectively.

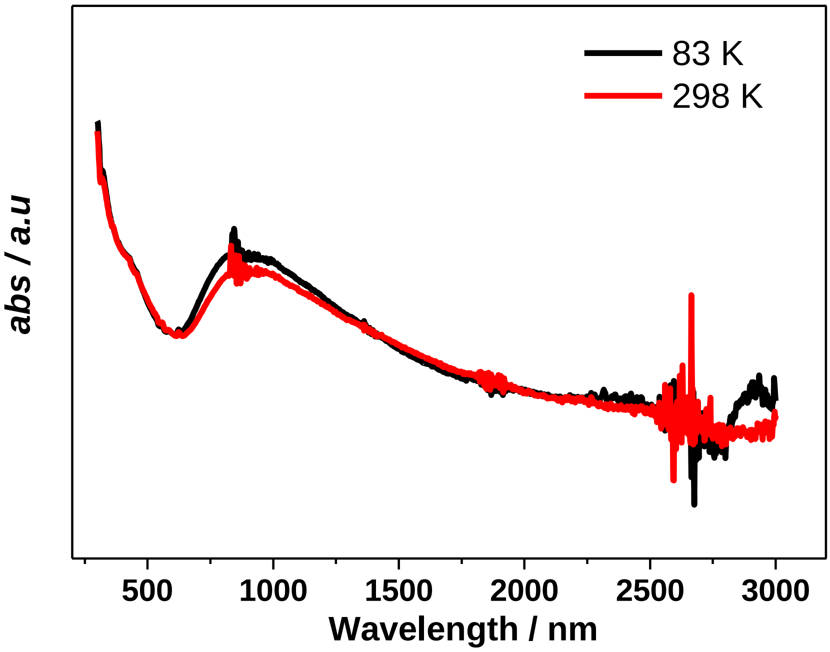

Supplementary Fig. 3 | Electronic absorption spectra of [Fe_{14}] at different temperatures. It should be noted that the broad character of the IVCT band in **[Fe_{14}]** exhibiting an extensive absorption tail is rather complicated when compared with typical mixed-valence complexes. This is due to this type of IVCT band frequently involving the superposition of two components, *i.e.*, adjacent IVCT (Fe^{II}-CN-Fe^{III}) and remote IVCT (Fe^{II}-NC-Fe^{II}-CN-Fe^{III}) bands as indicated in the reported cyanide-bridged systems with N-terminal mixed-valence state.[1-4] Hence, through analysis to obtain convincing information about the valence electron delocalization based on Hush theory is avoided.[5]

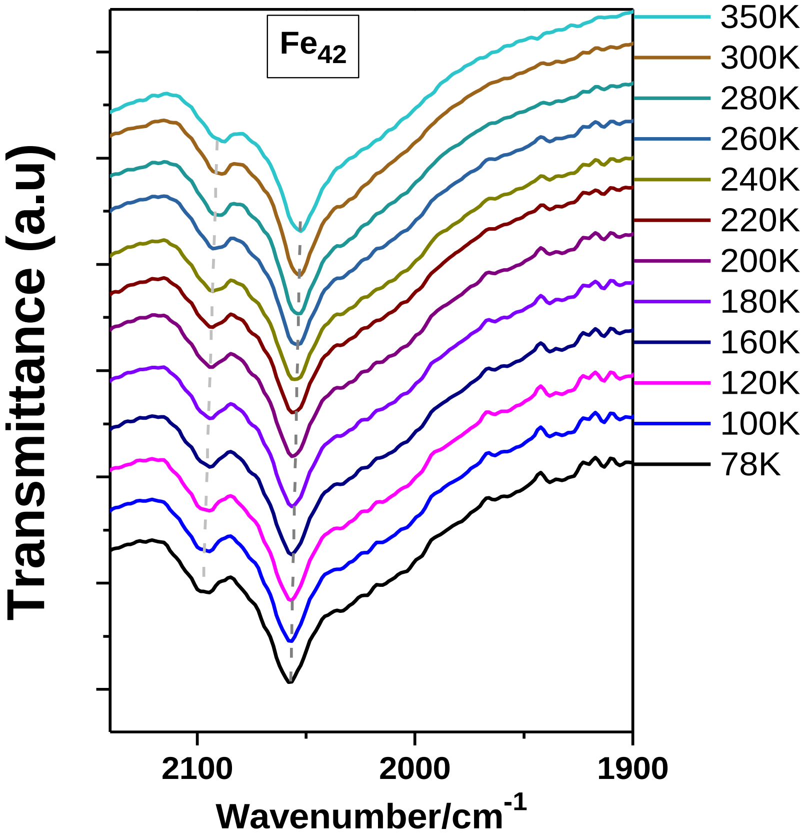

Supplementary Fig. 4 | *In-situ* infrared spectra of [Fe_{42}] recorded at different temperatures in the solid state. The **[Fe_{42}]** cluster only comprises the valence-trapped $Fe^{II\text{-}ls}$–CN–$Fe^{III\text{-}hs}$ structures.[6]

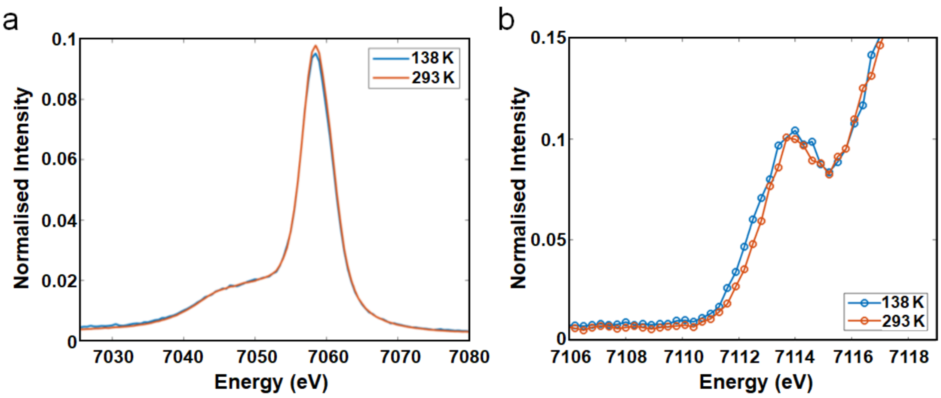

Supplementary Fig. 5 | Kβ emission spectroscopy and high-resolution K-edge XANES measurement. a, Kβ measurements of **[Fe_{14}]** at 138 and 293 K. b, K-edge XANES measurements at 138 and 293 K.

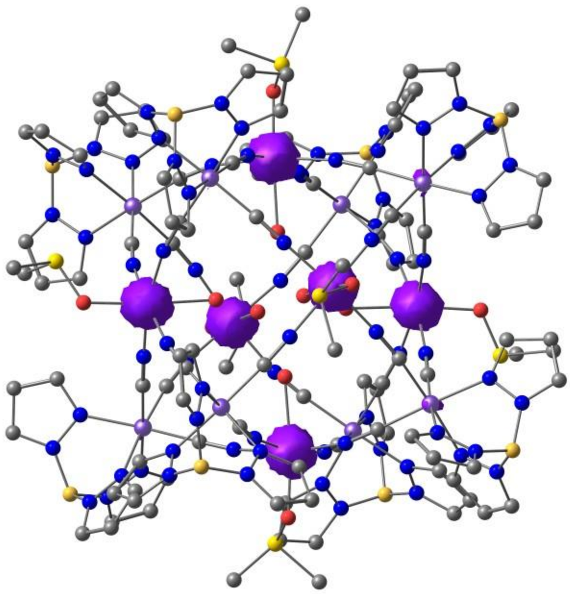

Supplementary Fig. 6 | Optimized atomic structure and spin density of [Fe_{14}]. The spin density (purple) of **[Fe_{14}]** with a total spin state of S = 14 is concentrated on the B-site Fe ions. The spin values of eight A-site Fe ions and six B-site Fe ions from Mülliken analysis were listed as following,

	Name	Fe(A)^1	Fe(A)^2	Fe(A)^3	Fe(A)^4	Fe(A)^5	Fe(A)^6	Fe(A)^7	Fe(A)^8
A - Site	Charge	2.85	2.85	2.85	2.85	2.85	2.85	2.85	2.85
	Spin	0.241	0.229	0.229	0.242	0.229	0.229	0.229	0.229

	Name	Fe(B)^1	Fe(B)^2	Fe(B)^3	Fe(B)^4	Fe(B)^5	Fe(B)^6
B-Site	Charge	2.69	2.69	2.69	2.69	2.69	2.69
	Spin	3.536	3.535	3.535	3.535	3.535	3.535

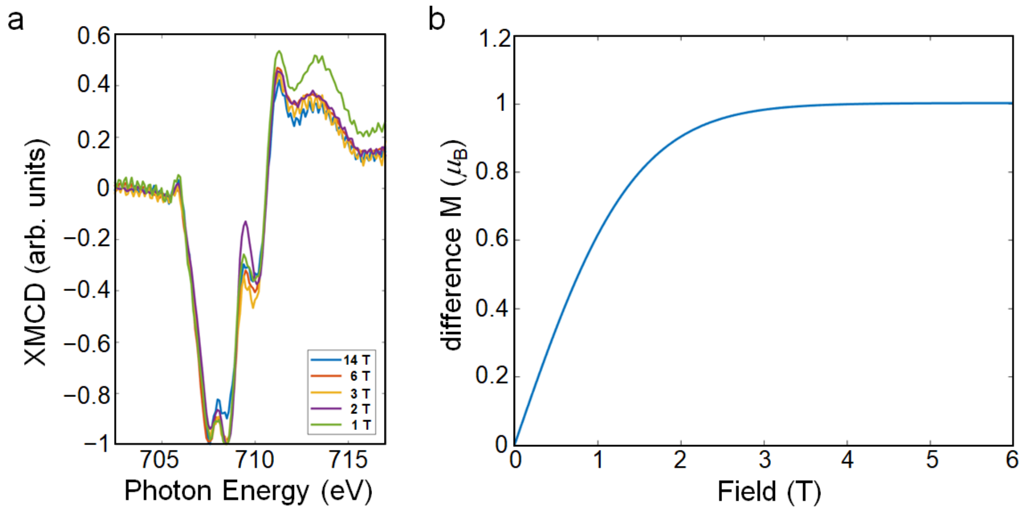

Supplementary Fig. 7 | X-ray magnetic circular dichroism measurements. a, **[Fe14]** L_3-edge XMCD spectra (3.5 K) at different applied fields normalized to −1. b, The 3.5 K Brillouin function difference for an $S = 5/2$ minus $S = 2$ indicating how the field dependence of the two spin moments varies by differing amounts in fields of less than 3 T. The spectra in **a** overlap for all applied fields indicting that the **[Fe14]** nanocube has a molecular spin ground state, where ferric and ferrous B sites are exchange coupled.

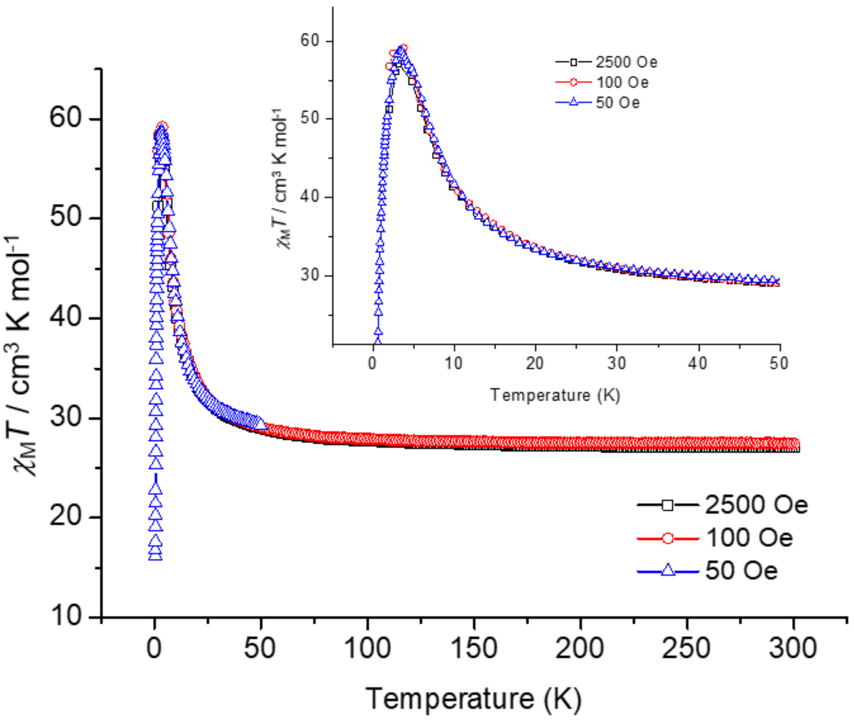

Supplementary Fig. 8 | Field-dependent magnetic data. The temperature dependence of $\chi_M T$ for **[Fe$_{14}$]** under magnetic fields of H_{dc} = 50, 100, and 2500 Oe. The inset depicts the enlarged plot at a low temperature. $\chi_M T$ under 50 Oe is measured between 0.5 and 50 K. $\chi_M T$ under 100 and 2500 Oe is measured between 2 and 300 K.

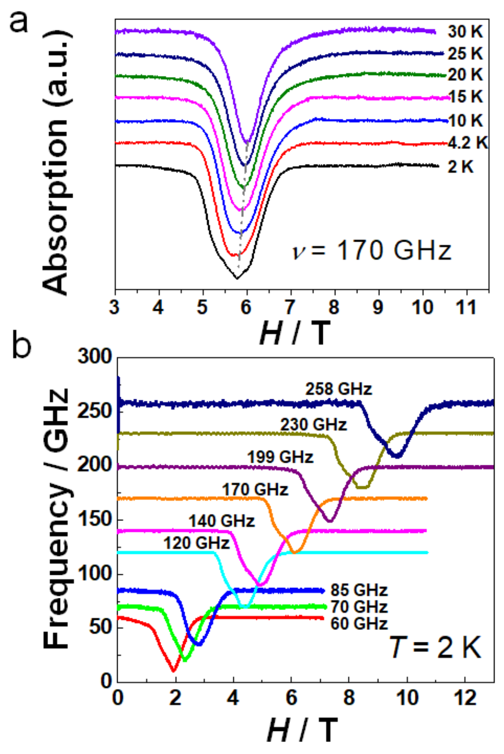

Supplementary Fig. 9 | High-frequency electron paramagnetic resonance spectra. a, Variable-temperature HF-EPR spectra of **[Fe$_{14}$]** at 170 GHz. b, Frequency-dependent EPR spectra of **[Fe$_{14}$]** at 2 K (60–258 GHz). At 2 K, the *g*~2.0 peaks exhibit the obvious Gaussian type line broadening and the small down shift of the resonance field in **b**, which become more resolved under the high-frequency and higher magnetic field. This feature would originate from the magnetic anisotropy due to the dipolar interactions in the rhombic lattice.

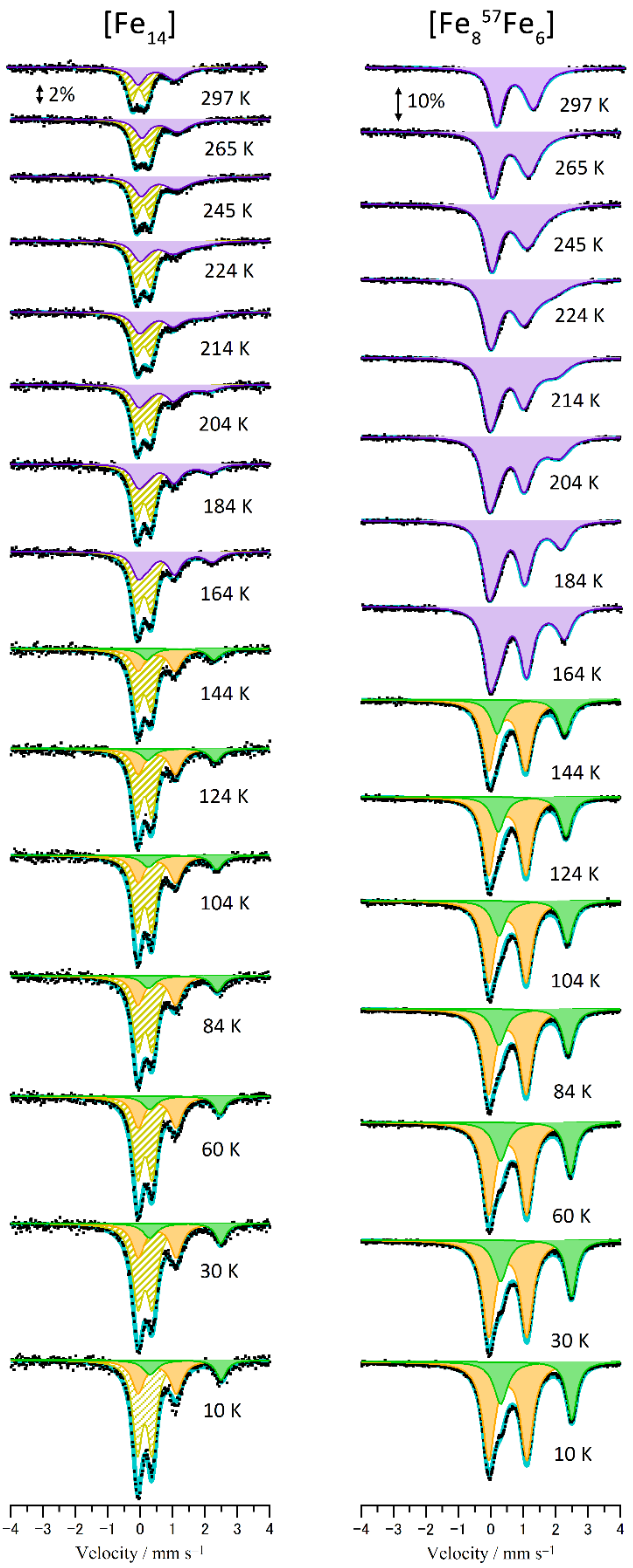

Supplementary Fig. 10 | Full zero-field ^{57}Fe Mössbauer spectra at all investigated temperatures. Variable-temperature ^{57}Fe Mössbauer spectra in the temperature range of 10–297 K at a zero-field for the natural isotopic **[Fe$_{14}$]** and the site-selected ^{57}Fe-enriched sample of **[Fe$_8$^{57}Fe$_6$]**. The purple profiles represent a merged component of Fe^{II}-*hs* and Fe^{III}-*hs* (B site) fitted using an electron hopping model. The striped yellow, orange, and green doublets correspond to Fe^{II}-*ls* (A site), Fe^{III}-*hs* (B site), and Fe^{II}-*hs* (B site), respectively.

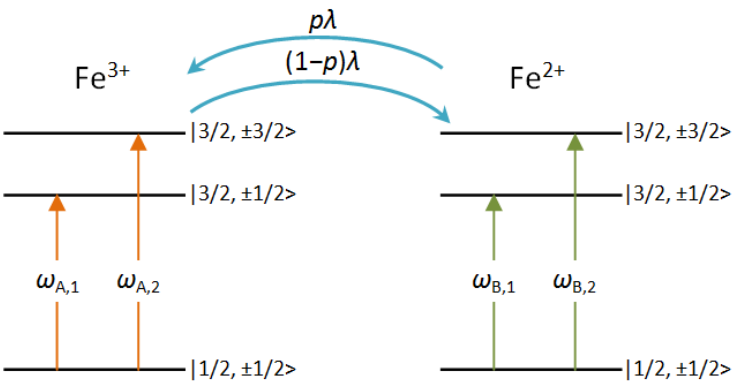

Supplementary Fig. 11 | Schematic illustration of electron hopping relaxation model.

Supplementary Table 1 | The crystal data of [Fe_{14}] at the different temperatures.

Temp.	25 K	123 K	300 K
Formula		$C_{108}H_{128}B_8Fe_{14}N_{72}O_{12}S_6$	
Formula Weight		3687.56	
Crystal System		trigonal	
Space group		*R*-3	
a [Å]	23.3915(5)	23.567(3)	23.802(3)
b [Å]	23.3915(5)	23.567(3)	23.802(3)
c [Å]	23.7244(7)	23.767(3)	23.715(5)
α [deg]		90	
β [deg]		90	
γ [deg]		120	
V [Å^3]	11242.0(8)	11432(4)	11635(4)
Z		3	
D(calc) [g cm^{-3}]	1.634	1.607	1.579
μ(MoKa) [mm^{-1}]	1.521	1.449	1.424
Crystal Size [mm]		0.12 × 0.16 × 0.21	
Tot. Data	25372	30157	31019
Uniq. Data	5727	4468	4529
R(int)	0.0663	0.069	0.032
Obs. data [$I > 2\sigma(I)$]	5132	4108	3934
R_1,[a] wR_2[b] [$I > 2\sigma(I)$]	0.0570, 0.1517	0.0347,0.0883	0.0304, 0.0786
R_1, wR_2 [all data]	0.0633, 0.1552	0.0373,0.0899	0.0363, 0.0808
GOF	0.978	1.07	1.08

[a]$R_1 = \sum||F_o| - |F_c||/\sum|F_o|$. [b]$wR_2 = \{\sum[w(F_o^2 - F_c^2)^2]/\sum[w(F_o^2)^2]\}^{1/2}$

Supplementary Table 2 | The selected bond distances (Å) and angles (°) for [Fe_{14}] at the different temperatures.

Temp.	25 K	123 K	300 K
Fe1-C16	1.873(4)	1.883(2)	1.882(2)
Fe1-N8	2.001(3)	2.007(2)	2.009(2)
Fe2-C13	1.864(4)	1.864(3)	1.871(3)
Fe2-C15	1.868(4)	1.872(3)	1.873(3)
Fe2-C14	1.874(4)	1.881(3)	1.876(2)
Fe2-N4	1.979(3)	1.996(2)	1.994(2)
Fe2-N2	1.999(3)	2.009(2)	2.013(2)
Fe2-N6	2.011(3)	2.015(2)	2.017(2)
Fe3-O2	2.014(3)	2.016(2)	2.016(2)
Fe3-N11	2.046(4)	2.066(2)	2.071(2)
Fe3-N12	2.058(4)	2.053(2)	2.054(2)
Fe3-N10	2.064(4)	2.068(2)	2.066(2)
Fe3-N9	2.074(3)	2.077(2)	2.076(2)
Fe3-O1	2.231(5)	2.262(2)	2.292(2)
C16a Fe1 C16b	87.58(17)	87.50(10)	87.67(10)
C16a Fe1 N8b	91.12(15)	91.16(9)	90.87(9)
C16b Fe1 N8b	94.55(15)	94.65(9)	94.74(9)
C16 Fe1 N8b	177.46(15)	177.42(9)	177.13(9)
C16a Fe1 N8a	94.55(15)	94.65(9)	94.74(9)
C16b Fe1 N8a	177.46(15)	177.42(9)	177.13(9)
C16 Fe1 N8b	91.12(15)	91.16(9)	90.87(9)
N8b Fe1 N8a	86.80(14)	86.75(9)	86.79(9)
C16a Fe1 N8	177.46(16)	177.42(9)	177.13(10)
C16a Fe1 N8	91.12(15)	91.16(9)	90.87(9)
C16 Fe1 N8	94.55(15)	94.65(9)	94.74(9)
N8b Fe1 N8	86.81(14)	86.75(9)	86.78(9)
N8a Fe1 N8	86.81(14)	86.75(9)	86.78(9)
C13 Fe2 C15	87.62(17)	87.88(11)	88.08(10)
C13 Fe2 C14	88.28(17)	88.41(11)	88.62(10)
C15 Fe2 C14	85.97(18)	86.19(10)	86.54(10)
C13 Fe2 N4	89.08(16)	89.18(10)	89.19(9)
C15 Fe2 N4	92.55(16)	92.64(10)	92.65(9)
C14 Fe2 N4	177.03(16)	177.35(10)	177.69(10)
C13 Fe2 N2	96.44(16)	96.36(10)	96.16(10)
C15 Fe2 N2	175.90(16)	175.73(10)	175.70(10)
C14 Fe2 N2	94.71(16)	94.38(10)	94.30(9)
N4 Fe2 N2	86.95(14)	86.97(8)	86.67(8)
C13 Fe2 N6	176.17(16)	176.34(10)	176.41(10)
C15 Fe2 N6	89.48(16)	89.38(10)	89.34(10)
C14 Fe2 N6	94.02(15)	93.84(10)	93.71(9)
N4 Fe2 N6	88.54(14)	88.52(8)	88.44(8)
N2 Fe2 N6	86.44(14)	86.36(9)	86.40(9)
O2 Fe3 N11c	96.67(15)	97.39(9)	97.64(9)
O2 Fe3 N12d	91.31(17)	91.50(10)	91.96(9)
N11a Fe3 N12b	171.85(17)	170.94(10)	170.30(9)
O2 Fe3 N10	92.65(15)	92.46(9)	92.92(9)
N11a Fe3 N10	88.28(14)	88.22(9)	88.37(9)
N12b Fe3 N10	89.72(15)	89.69(9)	89.78(9)
O2 Fe3 N9	92.60(14)	93.07(9)	93.69(9)
N11a Fe3 N9	91.26(14)	89.99(9)	91.02(9)
N12b Fe3 N9	90.01(14)	91.24(9)	89.73(9)
N10 Fe3 N9	174.75(15)	174.47(9)	173.38(9)
O2 Fe3 O1	177.03(18)	177.51(10)	178.09(9)
N11a Fe3 O1	86.24(17)	86.06(10)	84.19(9)
N12b Fe3 O1	85.80(18)	85.06(10)	87.67(10)
N10 Fe3 O1	88.03(18)	88.02(10)	86.22(10)
N9 Fe3 O1	86.72(17)	86.46(10)	85.71(10)

symm.code: a: *x*+*y*, -*x*+1, *z*; b: 1–*y*, 1+*x*–*y*, *z*; c: *x*–*y*+2/3, *x*+1/3, -*z*+4/3; d: *y*–1/3, -*x*+*y*+1/3, –*z*+4/3;

Supplementary Table 3 | The Mössbauer fitting parameters for natural isotopic [Fe_{14}].

			Fe^{II}-*ls* (Fraction: 57.1%; Site A)			Fe^{III}-*hs* (Fraction: 28.6%; Site B)[a]			Fe^{II}-*hs* (Fraction: 14.3%; Site B)[a]			
	Temp. / K	χ^2	IS / mm s^{-1}	QS / mm s^{-1}	LW / mm s^{-1}	IS / mm s^{-1}	QS / mm s^{-1}	LW / mm s^{-1}	IS / mm s^{-1}	QS / mm s^{-1}	LW / mm s^{-1}	Relax. Rate / Mrad s^{-1}
doublet×1 + electron hopping relaxation model	297	1.230	0.067(3)	0.441(6)	0.340(8)	0.262	0.971	0.335	1.343	1.503	0.335	187
	265	1.093	0.071(3)	0.444(5)	0.346(7)	0.286	0.981	0.335	1.343	1.502	0.335	130
	245	1.285	0.091(3)	0.441(4)	0.347(6)	0.298	1.003	0.335	1.343	1.550	0.335	92
	224	1.240	0.091(3)	0.443(4)	0.351(6)	0.339	1.055	0.335	1.340	1.691	0.335	54
	214	1.315	0.105(3)	0.439(4)	0.403(7)	0.395	1.098	0.354	1.292	1.771	0.354	33.3
	204	1.190	0.106(3)	0.439(4)	0.366(7)	0.424	1.117	0.374	1.275	1.835	0.374	23.1
	184	1.124	0.110(3)	0.442(5)	0.361(7)	0.467	1.132	0.420	1.245	1.935	0.420	10.1
	164	1.234	0.115(2)	0.444(4)	0.380(6)	0.484	1.141	0.419	1.253	1.985	0.419	6.1
doublet×2 + asym. doublet×1	144	1.124	0.119(3)	0.442(4)	0.379(9)	0.491	1.142	0.439	1.240	2.083	0.477/0.476	–
	124	1.353	0.127(2)	0.454(4)	0.365(6)	0.495	1.157	0.398	1.273	2.090	0.480/0.431	–
	104	1.359	0.129(2)	0.450(3)	0.347(5)	0.5115	1.150	0.448	1.304	2.117	0.505/0.421	–
	84	1.202	0.147(2)	0.449(4)	0.385(6)	0.5052	1.1589	0.449	1.3177	2.133	0.510/0.415	–
	60	1.432	0.145(2)	0.446(3)	0.373(5)	0.5216	1.1677	0.446	1.375	2.158	0.517/0.385	–
	30	1.463	0.148(2)	0.446(3)	0.404(5)	0.5221	1.1726	0.451	1.392	2.187	0.511/0.377	–
	10	1.503	0.138(2)	0.448(3)	0.348(5)	0.5211	1.1768	0.457	1.400	2.196	0.516/0.376	–

[a]All of the parameters of these species were fixed at the value obtained for the site-selected ^{57}Fe-enriched sample of [$\mathbf{Fe_{14}}$].

Supplementary Table 4 | The Mössbauer fitting parameters for the site-selected ^{57}Fe-enriched sample of [$Fe_8{}^{57}Fe_6$].

			Fe^{III}-*hs* (Fraction: 66.7%; Site B)			Fe^{II}-*hs* (Fraction: 33.3%; Site B)			
	Temp / K	χ^2	IS / mm s^{-1}	QS / mm s^{-1}	LW / mm s^{-1}	IS / mm s^{-1}	QS / mm s^{-1}	LW / mm s^{-1}	Relax. Rate / Mrad s^{-1}
doublet×1 + electron hopping relaxation model	297	3.001	0.262(3)	0.971(6)	0.335[a]	1.343[a]	1.503(12)	0.335[a]	187(4)
	265	2.132	0.286(4)	0.981(7)	0.335[a]	1.343[a]	1.502(12)	0.335[a]	130(3)
	245	2.264	0.298(4)	1.003(6)	0.335[a]	1.343[a]	1.550(10)	0.335[a]	92(2)
	224	2.832	0.339(8)	1.055(4)	0.335(14)	1.340(2)	1.691(10)	0.335(14)	54(3)
	214	4.071	0.395(4)	1.098(3)	0.354(9)	1.292(11)	1.771(8)	0.354(9)	33.3(12)
	204	3.044	0.424(3)	1.117(3)	0.374(8)	1.275(9)	1.835(8)	0.374(8)	23.1(10)
	184	4.032	0.467(2)	1.132(2)	0.420(5)	1.245(5)	1.935(5)	0.420(5)	10.1(5)
	164	3.027	0.484(2)	1.141(2)	0.419(6)	1.253(5)	1.985(6)	0.419(6)	6.1(6)
doublet×2 + asym. doublet×1	144	3.401	0.5007(1)	1.137(2)	0.478(3)	1.240(2)	2.083(4)	0.477/0.476	
	124	4.010	0.5077(1)	1.148(2)	0.446(3)	1.273(2)	2.090(4)	0.480/0.431	
	104	3.775	0.5115(9)	1.150(2)	0.448(2)	1.304(2)	2.117(4)	0.505(6)/0.421(3)	
	84	5.129	0.5052(7)	1.1589(13)	0.449(2)	1.318(2)	2.133(3)	0.510(6)/0.415(3)	
	60	3.804	0.5216(8)	1.1677(14)	0.446(2)	1.375(2)	2.158(3)	0.517(6)/0.385(3)	
	30	4.445	0.5221(6)	1.1726(12)	0.451(2)	1.392(2)	2.187(3)	0.511(5)/0.377(3)	
	10	4.133	0.5211(6)	1.1768(12)	0.457(2)	1.400(2)	2.196(3)	0.516(5)/0.376(3)	

[a]These parameters were fixed at the value obtained for the spectrum at the nearest temperature.

Supplementary method

Mössbauer analysis using electron hopping relaxation model

For spectral fitting of electron hopping relaxation, a model embedded into a MossWinn 4.0 software was applied, which is based on the theory and formalism of time-dependent exchange perturbations reported by Blume.[7] This model can be regarded as analogous to the relevant phenomena on nuclear magnetic resonance. A simplified mathematical representation was given by Wickman,[8] which is introduced into the references on electron hopping observed in FeOCl intercalation compounds[9] and trinuclear acetate-bridged [$Fe^{II}Fe^{III}_2$] complexes.[10] Absorption intensity $I(\omega)$ for a system showing two nuclear transitions at resonant frequencies of ω_A and ω_B, where the respective probabilities are p and $(1-p)$, is given by the following equation (1):

$$I(\omega) = \frac{(1+\tau\Gamma)P + QR}{P^2 + R^2}, \quad (1)$$

where

$$P = \tau\left[\Gamma^2 - \left\{\frac{1}{2}(\omega_A + \omega_B) - \omega\right\}^2 + \frac{1}{4}(\omega_A - \omega_B)^2\right] + \Gamma, (2)$$

$$Q = \tau\left[\frac{1}{2}(\omega_A + \omega_B) - \omega - \frac{1}{2}\big(p - (1-p)\big)(\omega_A - \omega_B)\right], (3)$$

$$R = \left[\frac{1}{2}(\omega_A + \omega_B) - \omega\right](1 + 2\tau\Gamma) + \frac{1}{2}\big(p - (1-p)\big)(\omega_A - \omega_B), (4)$$

The line width represents Γ, and the relaxation time τ is defined as the reciprocal (λ^{-1}) of the relaxation rate, where $p\lambda$ corresponds to the transition rate. In our case, relaxation processes occur between Fe^{2+} and Fe^{3+} of the same $|I, m_I>$ state ($\omega_{A,1} \leftrightarrow \omega_{B,1}$ and $\omega_{A,2} \leftrightarrow \omega_{B,1}$) as shown in Supplementary Figure 11. The total intensity, $I_{total}(\omega)$, is given in eq 5:

$$I_{\mathrm{total}}(\omega) = \sum_{i=1}^{2} I_i(\omega) = \sum_{i=1}^{2} \frac{(1+\tau\Gamma)P_i + Q_i R_i}{P_i^2 + R_i^2}, (5)$$

where P_i, Q_i, and R_i correspond to eqs 2, 3, and 4 replaced with the terms of ω_{A},i and ω_{B},i instead of ω_A and ω_B, respectively.

Supplementary References:

1. D'Alessandro, D. M. & Keene F. R. Intervalence Charge Transfer (IVCT) in Trinuclear and Tetranuclear Complexes of Iron, Ruthenium, and Osmium. *Chem. Rev*. **106**, 2270 − 2298 (2006).
2. Nihei, M., Ui, M., Hoshino, N. & Oshio, H. Cyanide-bridged iron(II,III) cube with multistepped redox behavior. *Inorg. Chem.* **47**, 6106–6108 (2008).
3. Oshio, H., Onodera, H., Tamada, O., Mizutani, H., Hikichi, T. & Ito, T. Cyanide-Bridged Fe−Fe and Fe−Co Molecular Squares: Structures and Electrochemistry of $[Fe_4^{II}(\mu\text{-}CN)_4(bpy)_8](PF_6)_4\cdot 4\,H_2O$, $[Fe_2^{II}Co_2^{II}(\mu\text{-}CN)_4(bpy)_8](PF_6)_4\cdot 3\,CHCl_3\cdot 2\,CH_3CN$, and $[Fe_2^{II}Co_2^{III}(\mu\text{-}CN)_4(bpy)_8](PF_6)_6\cdot 2\,CHCl_3\cdot 4\,CH_3NO_2$. *Chem. Eur. J.*, **6**, 2523–2530 (2000).
4. Oshio, H., Onodera, H. & Ito, T. Spectroelectrochemical Studies on Mixed-Valence States in a Cyanide-Bridged Molecular Square, $[Ru^{II}{}_2Fe^{II}{}_2(\mu\text{-}CN)_4(bpy)_8](PF_6)_4\cdot CHCl_3\cdot H_2O$. *Chem. Eur. J.* **9**, 3946–3950 (2003).
5. Hush, N. S. Intervalence-transfer absorption. Part 2. Theoretical considerations and spectroscopic data. *Prog. Inorg. Chem.* **8**, 391–444 (1967).
6. Kang, S. *et al.* A ferromagnetically coupled Fe_{42} cyanide-bridged nanocage. *Nature Commun*. **6**, 5955−5960 (2015).
7. Blume, M. Stochastic theory of line shape: Generalization of the Kubo-Anderson model. *Phys. Rev.* **174**, 351–358 (1968).
8. Herber, R. H. & Eckert, H. Electron hopping in FeOCl intercalation compounds: A Mössbauer relaxation study. *Phys. Rev. B* **31**, 34–41 (1985).
9. Dziobkowski, C. T., Wrobleski, J. T. & Brwon, D. B. Magnetic and Spectroscopic Properties of $Fe^{II}Fe^{III}{}_2O(CH_3CO_2)_6L_3$, L = H_2O or C_5H_5N. Direct Observation of the Thermal Barrier to Electron Transfer in a Mixed-Valence Complex. *Inorg. Chem.* **20**, 679–684 (1981).
10. Wickman, H. H., Klein, M. P. & Shirley, D. A. Paramagnetic Hyperfine Structure and Relaxation Effects in Mössbauer Spectra: Fe^{57} in Ferrichrome *A. Phys. Rev.* **152**, 345–357 (1966).
